# Child Mental Health Research in Low- and Middle-Income Countries: A Twin-Family Feasibility Study in Nigeria

**DOI:** 10.1007/s10519-025-10235-z

**Published:** 2025-10-13

**Authors:** Olakunle Ayokunmi Oginni, Olatokunbo Oguns, Olusola Jeje, Oluwatosin Olorumoteni, Boladale Mapayi, Ruth Gilbert, Dan J. Stein, Frühling Rijsdijk, Anita Thapar

**Affiliations:** 1 Wolfson Centre for Young People’s Mental Health and Centre for Neuropsychiatric Genetics and Genomics, Division of Psychological Medicine and Clinical Neuroscience, Cardiff University, Cardiff, CF24 4HQ UK; 2 Department of Haematology, Obafemi Awolowo University, Ile-Ife, Nigeria; 3 Department of Chemical Pathology, Obafemi Awolowo University, Ile-Ife, Nigeria; 4 Department of Paediatrics, Obafemi Awolowo University, Ile-Ife, Nigeria; 5 Department of Mental Health, Obafemi Awolowo University, Ile-Ife, Nigeria; 6 University College London Institute of Child Health, University College London, London, UK; 7 SAMRC Unit on Risk and Resilience in Mental Disorders, Department of Psychiatry and Neuroscience Institute, University of Cape Town, Cape Town, South Africa; 8 Department of Psychology, Anton de Kom University, Paramaribo, Suriname

**Keywords:** Childhood mental health, Genetics, Twin-family research, Low- and middle-income (LAMI) countries, Participant involvement and engagement (PIE)

## Abstract

Globally, most children and adolescents live in low- and middle-income (LAMI) countries. Despite the high and under-recognized mental health burden in these settings, there is little systematic research to inform cost-effective mental health interventions. Identifying causal risk and protective mechanisms is important to inform such interventions. Longitudinal genetically informative designs can help identify potentially causal environmental mechanisms in the etiology of childhood psychopathology but few have been carried out in LAMI settings. We tested the feasibility of a twin-family study in a semi-urban setting in South-Western Nigeria. We recruited 320 family units, each comprising at least one parent and both twins aged 2.5–5.9 (x̄ = 4.0 ± 0.92) years from two towns using five strategies: direct and indirect contacts, radio adverts, cluster sampling (based on local administrative units) and snowball sampling. Participants were asked about their willingness to participate in future research including providing biological samples. These were supplemented with participant engagement activities before and after data collection. Snowball sampling was the most effective strategy while cluster sampling was the least effective (recruiting 46.3% and 8.3% of participants, respectively). Direct contacts and cluster sampling appeared prone to excluding under-represented participants. A large proportion of the participants (98–99%) were willing to participate in future studies. Challenges included grant administration (finance and ethical priorities) and desirability bias. Twin research is feasible in LAMI sub-Saharan Africa, with snowball sampling being an efficient means of recruiting a diverse sample. Participant involvement and engagement is useful to inform the execution of genetically-informative research in a LAMI context.

**Supplementary Information:**

The online version contains supplementary material available at 10.1007/s10519-025-10235-z.

## Introduction

Low- and middle-income (LAMI) countries currently contribute 80% of the global burden of mental health (Thyloth et al. 2016) which is increasing globally (GBD 2019 Mental Disorders Collaborators 2022). In 50% of cases, adult psychopathology emerges before age 14 years (Solmi et al. 2022); hence, childhood and adolescence are critical periods for mental health risk. This reflects ongoing brain development and maturation during childhood and adolescence as well as major social transitions (Lupien et al. 2009). Currently, 1 out of every 6–8 children in the region experience common psychopathology such as emotional and behavioral difficulties (Cortina et al. 2012; Jörns-Presentati et al. 2021). Fifty percent of the population in sub-Saharan Africa consists of those younger than 18 years (Galal 2024) and the region is projected to emerge as the most populous by 2100 (United Nations Department of Economic and Social Affairs Population Division 2023). In view of these, sub-Saharan Africa is set to emerge as the region with the highest burden of child and adolescent psychopathology globally.

This high burden is largely unmet: on the one hand, mental health services are generally underfunded; on the other hand, child and adolescent mental health needs are less recognized and prioritized compared to adult mental health (Zhou et al. 2020). To illustrate, of the 250 psychiatrists serving Nigeria’s population of 228 million (Fadele et al. 2024; World Health Organization 2024), less than a quarter are child and adolescent psychiatrists (personal communication) while half the population is less than 18 years (UNICEF 2023). Thus, the often-cited index of unmet mental health need in Nigeria (psychiatrist to population ratio of 1:1,000,000; World Health Organization 2021) is at least 4 times higher among children and adolescents (1:2,280,000) compared to adults (1:570,000).

The high unmet need and scarce resources in this region (Zhou et al. 2020) suggest early preventive interventions as the most cost-efficient strategy in tackling the rising burden of child and adolescent psychopathology in sub-Saharan Africa. However, there is little research to inform such interventions (Owen et al. 2016; Zhou et al. 2020). Studies from high-income countries (HICs) implicate a wide variety of risk factors such as parent-/child-based biological (Hysing et al. 2007; Kuhlman et al. 2020; Neumann et al. 2022) and psychosocial risk factors (Greene et al. 2018; Hughes et al. 2017; Marsh et al. 2020; Peverill et al. 2021). Biological risk factors include genetic risk, pregnancy- and delivery-related complications, malnutrition, inflammation and chronic physical illnesses (Fasesan et al. 2023; Hysing et al. 2007; Neumann et al. 2022). Social and environmental risk factors include poverty, parental mental illness, domestic violence, chaotic home environments, and emotional/physical abuse and neglect of the child, other types of victimization, exposure to conflict and war, as well as displacement (Economic Commission for Africa 2021; Greene et al. 2018; Hughes et al. 2017; Marsh et al. 2020; Peverill et al. 2021).

This research gap is critical considering the vulnerability of the developing brain (Lupien et al. 2009) and the childhood/adolescence onset of most psychopathology (Solmi et al. 2022). Also, relatively little is known about the role of genetic predisposition in LAMI contexts (Fatumo et al. 2022) and interplay with environmental risk factors specific to resource-poor contexts (Oginni & Hur 2024). These risk factors include severe poverty, malnutrition, higher mental health stigma (Adewuya and Makanjuola 2008; Zhou et al. 2020) and child labor. Finally, the role of general and contextual protective factors in early life such as parental social connectedness, social and economic support, parent warmth and religious beliefs (Bowes et al. 2010; Kasen et al. 2012; Osundina et al. 2017) deserves further investigation. Identifying protective and risk factors and their mechanisms is critical for developing context-relevant preventive public mental health programmes (Oginni et al. 2024; Zhou et al. 2020). These can include promoting protective factors and innovatively building alliances with locally relevant social agencies like religious institutions.

Longitudinal twin-family designs are well-suited to identifying potential causal effects of environmental risks (Willoughby et al. 2023) and their interplay with genetic risk (Thapar and Rutter 2019). However, most of these studies are typically conducted in HICs (Hur et al. 2019a, b) and the findings may not generalize to sub-Saharan Africa due to e.g., different cultural contexts (Renwick et al. 2024) and scarce resources (World Health Organization 2021). Currently, the only twin studies in LAMI settings are a hospital-based sample in Guinea Bissau (Bjerregaard-Andersen et al. 2019), a school-based adolescent sample in Nigeria (Hur, et al. 2019a, b) and a population-ascertained adult cohort in Sri Lanka (Jayaweera et al. 2018). These are limited in the context of developmental psychopathology by being focused on metabolic phenotypes and adult/adolescent psychopathology, being cross-sectional and not incorporating the children’s family contexts. In turn, these mean that early sensitive developmental periods and risk/causal mechanisms are not investigated. Furthermore, recruitment strategies in Guinea-Bissau and Nigeria may yield non-representative samples while that in Sri-Lanka which used the electoral register was representative (Sribbadana et al. 2008). The latter approach may not be feasible in Nigeria where birth registration and electoral participation are low (Federal Ministry of Health and Social Welfare of Nigeria 2024; Adigun 2020). Although electoral registration is high, the register may be inaccurate as it has not previously been audited (Este 2023). Importantly, the biases of the other approaches have not been previously examined. Considering this knowledge gap, the present pilot study investigated the feasibility of carrying out twin-family research among families with young twins in a rural setting in Nigeria – the most populous country in sub-Saharan Africa (Worldometer 2025), including the relative performance of different recruitment strategies.

## Methods

### Study Site

**Fig. 1:**
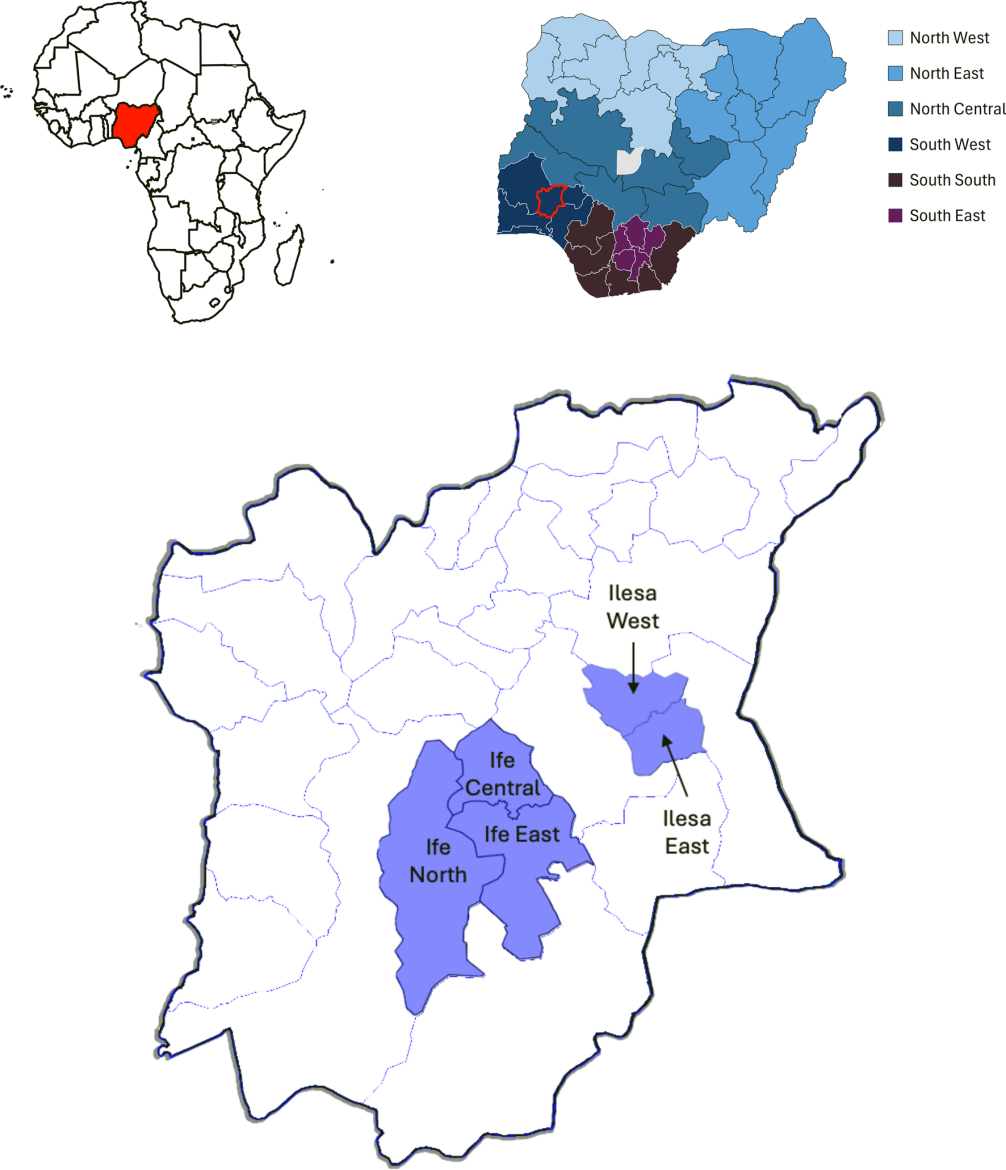
Map of Osun state with study sites highlighted: Ife (comprising 3 local government areas: Ife Central, Ife East and Ife North) and Ilesa (comprising 2 local government areas: Ilesa West and Ilesa East). *Insets*: Map of Africa with Nigeria highlighted and Map of Nigeria with 36 constituent states organized into geopolitical zones and Osun highlighted in Southwest geopolitical zone

The study was carried out in two semi-urban towns—Ile-Ife and Ilesa which are located in Osun state in the Southwest geopolitical zone (Fig. 1). In the country, the zone is one of the most politically stable, has one of the highest twinning rates (up to 46 twins/1000 births in the country Akinboro et al. 2008; Smits and Monden 2011) and one of the lowest under-5 mortality rates (42/1000, compared with 140/1000 live births in the North-West zone; Federal Ministry of Health and Social Welfare of Nigeria 2024).

### Sample

We recruited households with twins aged between 3 and 6 years with each household comprising at least one parent and one twin pair over a period of 14 weeks. We originally set out to recruit only families in which both parents were present but included single-parent households towards the end of the study to increase the diversity of the sample.

### Recruitment (Details of Each Strategy are Provided in Table 1)

**Table 1:** Comparison of strategies to recruit households with twins in Nigeria

Strategy and description	Advantages	Limitations	Overcoming limitations in the present study
i. Direct contact based on an existing register derived from health (birth and vaccination) records and previous research (Hur et al. 2024; Odintsova et al. 2018; Oginni et al. 2024) Telephone calls were made to potential participants by research assistants to invite them and schedule appointments. Weekly text messages were further sent to remind those who had not yet participated, to thank those who had participated and ask families to inform other families with twins known to them Proportion contributed to total sample: 24.1%	• Twin births in hospitals were objectively and reliably ascertained • Pregnancy- and delivery-related details could be obtained from hospital-based birth records	• Identified families may be non-representative due to: – Majority (57%) of deliveries in Nigeria occur outside the hospital (Federal Ministry of Health and Social Welfare of Nigeria, 2024) with high-income families potentially being over-represented among the hospital births due to private healthcare financing in Nigeria (Madu & Osborne 2023) – Over-representation of complicated pregnancies and deliveries in hospitals with inflation of the proportion of twins with pregnancy-/delivery-related mortality and morbidity • Poor maintenance of hospital records with contact details being outdated. In a prior study, only 31.7% of mothers identified from hospital records were contactable and available to participate in research (Oginni et al. 2024). Other families had moved away or changed their phone numbers while some twins had died	• Alternative strategies were used as a supplement for recruitment • Sociodemographic characteristics of the families were compared across the different strategies
ii. Recruitment through radio adverts Daily adverts were aired on radio in the study sites 4 weeks prior to commencing the study and throughout the duration of data collection. Phone numbers were included in the advert so that families could contact the research team to schedule appointments The adverts were supplemented by radio talks in Yoruba discussing childhood mental health and the significance of twins for genetic research Proportion contributed to total sample: 23.6%	• Quick method for reaching a large number of potential participants within a short time • Can be cost-effective in larger studies by shortening the duration of recruitment	• The sample may be non- representative with risk for selection bias • Rigor would be required to ascertain the eligibility of participants (Oginni et al. 2024) • There are many radio stations per state in Nigeria and preliminary work will be required to determine which stations were more popular among the target population	• Alternative strategies were used as a supplement for recruitment • Sociodemographic characteristics of the families were compared across the different strategies • Eligibility of participants was ascertained using multiple levels of screening including inspecting parents’ names as written on the twins’ birth certificates and on parents’ photographic identification cards, and photographs depicting parents and twins (Oginni et al. 2024) • During pre-study PIE activities, participants recommended radio over television for advertising the study. We also previously ascertained the which radio stations were most popular among participants and used these in the present study (Oginni et al. 2024)
iii. Multi-stage cluster sampling The study sites (Ile-Ife and Ilesa) were divided into 38 and 35 clusters (wards) respectively. Each cluster was subdivided into units comprised of landlord-tenants associations which were each headed by a chairperson. Ten and 6 clusters were selected in Ife and Ilesa respectively, and all the chairpersons of each landlord-tenant association in the selected cluster were approached and requested to help identify families with twins in their communities^a^ Proportion contributed to total sample: 8.3%	• Can enable a more representative sample • Can provide opportunity to recruit less-represented participants e.g., those residing in rural areas • Considering the traditional significance of twin births in southwestern Nigeria (Leroy et al. 2002; Olupona 2023), cluster leaders will be able to identify households with twins	• Depends on the goodwill and receptiveness of the chairpersons of the landlord-tenants associations which can vary from association to association • Parents of twins may be unwilling for their contact details to be shared with the research team	• Sociodemographic characteristics of the families were compared across the different strategies • We established credibility and rapport with chairpersons based on our collaboration with Nigerian institution as recommended during PIE activities
iv. Indirect contacts via primary schools, markets, churches and mosques Primary schools were identified and headteachers were informed about the study and requested to inform parents Churches and mosques were identified in the study sites and senior clerics were informed about the study and requested to inform their congregation about the study Announcements were in made in the markets Proportion contributed to total sample: 22.1%	• Involving religious leaders may make participants more willing to engage with the study given the high rate of religious affiliation in Nigeria (> 99%) (Federal Ministry of Health and Social Welfare of Nigeria 2024). This approach may also provide opportunities for future collaborations • High traffic in market may facilitate rapid dissemination of information regarding the study	• The official age of enrollment in primary schools in Nigeria is 6 years (Federal Republic of Nigeria 2014), hence, primary schools may not be optimal for recruiting a younger age group as was highlighted during pre-study PIE (Oginni et al. 2024) • While religious leaders were supportive, we initially struggled to access the mosques and this may be reflective of more conservative gender roles in Islam which can be reinforced by the sociocultural context (Öztürk 2023) • Market announcements were discontinued quickly as the cost outstripped the number of participants recruited using this approach. We targeted peak periods to maximize our reach but it is possible that buyers and sellers could not pay attention to us due to ongoing transactions	• Despite the official age of primary school enrollment, children often enroll in primary school before the age 6 years (Sasu 2022) so this approach was still useful • The principal investigator (male) identified a Muslim cleric who was a father of twins and had participated in previous twin research, and facilitated an introduction to senior Muslim clerics in one of the study sites (Ile-Ife) • Market announcements were targeted for the mornings of ‘market days’ when business is at a peak. Future approaches may target markets on non-market days when business is relatively less and off-peak periods later in the day when traders may be more relaxed
v. Snowball sampling Families identified from the earlier strategies were requested to invite other families with twins to participate in the study. Traditional birth attendants^b^ were also requested to invite mothers of twins in the community Proportion contributed to total sample: 46.3%	• Potentially quick and effective recruitment strategy given popularity of twins in southwestern Nigeria (Leroy et al. 2002; Olupona 2023) and high rate of out-of-hospital deliveries (Federal Ministry of Health and Social Welfare of Nigeria 2024) • During pre-study PIE, public contributors and traditional birth attendants reported knowing other mothers of twins and were willing to help recruit other households for the proposed study (Oginni et al. 2024)	• Sample may not be representative of the general population but this has not been tested	• Alternative strategies were used as a supplement for recruitment • Sociodemographic characteristics of the families were compared across the different strategies

*PIE* participant involvement and engagement

^a^For administration, each state in Nigeria is divided into Local Government Areas, each of which is further subdivided into wards. Communities in urban and suburban regions in Nigeria are locally organized into associations of houseowners (landlords) and tenants living within defined zones and may comprise 20–50 households headed by a chairman (Owojori and Solarin 2020)

^b^Traditional birth attendants are community-based midwives who supervise deliveries in community—at home or faith-based organizations

We used five strategies to identify and recruit families with twin children: i. Directly contacting parents of twins who had previously been identified from a register based on healthcare records and previous research; ii. Radio adverts; iii. Multi-stage cluster sampling; iv. Indirect contact through primary schools, markets and churches/mosques; and v. Snowball sampling. Pre-recruitment Participant Involvement and Engagement (PIE) have been described elsewhere (Oginni et al. 2024).

### Procedure (Table 1)

Radio adverts and fliers were circulated during indirect outreach and given to participating parents. These included dedicated phone numbers for interested participants to make enquiries and register their interest. Mothers of twins identified from hospital records and previous research were contacted by phone calls and weekly text messages. The initial telephone contacts were used to preliminarily screen and consent potential participants. Based on feedback from pre-recruitment PIE (to facilitate trust in potential participants and allay safety concerns; Oginni et al. 2024), data were collected in dedicated offices in teaching hospitals affiliated with the collaborating institution in Nigeria. When families arrived for the study, a second line of screening was carried out to confirm i. twins’ ages by inspecting their birth certificates, ii. parents’ identities by inspecting photo IDs (such as the National Identity cards or Driver’s licences), and iii. parent–child relationships by checking that the names of parents as stated on the birth certificates matched those stated on their identification documents, and inspecting a non-recent photograph including the twins and their parents.

Questionnaires were administered face-to-face by research assistants based on recommendations from pre-recruitment PIE. These comprised i. a sociodemographic and feasibility section including how the participants heard about the study, willingness to participate in future research and provide blood and saliva samples in the current and future studies and their preferred venue for data collection in a future study; ii. a parent-based section and iii. a parent-reported child-based section; both for psychopathology and risk/protective factors (Supplementary Table 1). All questionnaires were translated using a modified WHO protocol (Kalfoss 2019): i. initial translation from English into Yoruba, ii. translated questionnaires were checked by two Psychiatrists fluent in Yoruba and English with recommendations, iii. updated translated questionnaires were discussed with members of the pre-recruitment PIE group (to ensure translated questions could be easily understood by non-technical readers), recommendations included rephrasing some words to facilitate comprehension and increase cultural acceptability to research participants, iv. approved Yoruba questionnaires were back-translated into English by a different translator; v. the back-translated English questionnaires were compared with original English versions to ensure that the meanings were preserved, vi. throughout the study, we recorded any difficulties participants had in comprehending the questions. Because of the sensitive nature of some of the questions, fathers were interviewed in separate rooms from the mothers.

Zygosity was determined using a direct question to parents about whether their twins were identical or non-identical and selected questions from the twin confusion section of the zygosity questionnaire (Goldsmith 1991). We further took photographs of 57 twin pairs, which were evaluated by a member of the team to determine the accuracy of the direct questions and the individual questionnaire items. Using this approach, we found that the mothers’ response to direct questions had a higher accuracy for determining the twins’ zygosity (correctly identifying 18 out of 20 monozygotic twins, 20 out of 22 for same-sex dizygotic twins and 15 out of 15 for opposite-sex dizygotic twins—90%, 91% and 100% respectively) compared to questions about similarity. Additionally, considering the familial aggregation of twinning in Southwestern Nigeria (Hur et al. 2024) and the prospect of recruiting parents as twins for future quasi-experimental research (Ahmadzadeh et al. 2022), we asked whether the parents or their siblings were twins. Thirty-eight (out of 334 parents who responded-11.4%) reported being a twin while 54 out of 312 (17.3%) had twin siblings.

We obtained anthropometric measurements (height and weight) for all parents and children using standard methods. To assess inflammatory markers and indices of malnutrition, and to test the feasibility of collecting biological samples in future research, blood samples were collected by a trained laboratory scientist from a subsample of 60 households. This was done in the hospital as advised during the pre-recruitment PIE to allay safety concerns (Oginni et al. 2024) and offered to the first families who were recruited. Of these, one family refused due to safety concerns, one father could not have his sample taken due to needle phobia and veins could not be found in two children due to subcutaneous fat. Blood samples were analysed in the hospital laboratories and all participants wanted to be informed about the results of the tests carried out. No samples were stored for future analyses.

Data collection took 120 to 150 min and parents were informed about this during the initial telephone contact and at the beginning of the study. Midway into the study, parents and children were provided with snacks and non-alcoholic drinks to facilitate their continued participation, while children were provided with balloons to prevent them from getting irritable or distracting their parents while waiting. Each complete family was paid £10 (N20,000; £5 [N10,000] when only one parent was present) to compensate for their time and travel, and each child was given a water-bottle.

Participant Involvement and Engagement (PIE): Pre-recruitment PIE findings have been described elsewhere (Oginni et al. 2024). Post-recruitment PIE was carried out after data collection, ten parents were invited to participate in a group discussion about their experience of the study, their motivation and perceived challenges to participation, perceived benefits for them and their children, suggestions for future studies and dissemination.

## Results

The total sample comprised 320 families of children aged 2.5–5.9 years (Mean = 4.0 ± 0.92), 190 and 130 families from Ile-Ife and Ilesa respectively. Both parents were available in 270 families while one parent was available for 50 families (49 mothers and 1 father). Among the participating households, 93 (29.1%) families had monozygotic twins (38 [40.9%] male and 55 female) and 222 (69.4%) dizygotic twins (57 same-sex male, 43 same-sex female and 122 opposite-sex—25.7%, 19.4% and 55.0% respectively), while 5 (1.56%) had triplets. On average, fathers were older than mothers (mean [SD] were 40.6 [7.57] and 34.1 [6.21] years, respectively; Table 2). Most of the parents were either married or cohabiting (97.6%), while the proportion of single parents was higher among mothers (4.1%) compared to fathers (0.4%). Most of the participants were Christians and had secondary or tertiary education.

**Table 2:** Parents’ sociodemographic characteristics by parents’ sex

Variables		Total		Father		Mother	
		n (589)^a^	%	n (270)	%	n (319)	%
*Sociodemographic*							
Parents age (years)	(Mean [SD])	37.1	[7.58]	40.6	[7.57]	34.1	[6.21]
Marital status	Single	14	2.4	1	0.4	13	4.1
	Cohabiting	93	15.8	38	14.1	55	17.2
	Married	482	81.8	231	85.6	251	78.7
Religion^b^	Christian	483	82.1	216	80.3	267	83.7
	Muslim	105	17.9	53	19.7	52	16.3
Education	Primary	43	7.3	22	8.1	21	6.6
	Secondary	296	50.3	133	49.3	163	51.1
	Tertiary	239	40.6	107	39.6	132	41.4
	Postgraduate	11	1.9	8	3.0	3	0.9
Income (₦,000)	(Median [range])	46.5	[0.0–5000.0]	390.0	[5.0–5000.0]	20.0	[0.0–900.0]
Household wealth	(Mean [SD])	4.9	[1.68]	5.3	[1.58]	4.6	[1.71]
Parental participation	One parent	50	8.5	1	0.4	49	15.4
	Both parents	540	91.5	269	99.6	270	84.6
*Feasibility*							
Preferred interview location	Home	144	24.4	44	16.3	100	31.3
	Hospital	445	75.6	226	83.7	219	68.7
Willingness to participate in future research	Yes	584	99.2	267	98.9	317	99.4
	No	5	0.8	3	1.1	2	0.6
Willingness to provide blood sample in future	Yes	578	98.1	263	97.4	315	98.7
	No	11	1.9	7	2.6	4	1.3
Willingness to provide saliva sample in future	Yes	580	98.5	266	98.5	314	98.4
	No	9	1.5	4	1.5	5	1.6
Donated blood in current study (n = 122)	Yes	119	97.5	59	96.7	60	98.4
	No	3	2.5	2	3.3	1	1.6
*Recruitment*							
Snowball	Yes	273	46.3	126	46.7	147	46.1
	No	316	53.7	144	53.3	172	53.9
Direct outreach	Yes	142	24.1	56	20.7	86	27.0
	No	447	75.9	214	79.3	233	73.0
Cluster sampling	Yes	49	8.3	44	16.3	5	1.6
	No	540	91.7	226	83.7	314	98.4
Radio adverts	Yes	139	23.6	73	27.0	66	20.7
	No	450	76.4	197	73.0	253	79.3
Indirect outreach	Yes	130	22.1	43	15.9	87	27.3
	No	459	77.9	227	84.1	232	72.7
Number of strategies	1	462	78.4	206	76.3	256	80.3
	2	110	18.7	56	20.7	54	16.9
	3	17	2.9	8	3.0	9	2.8

With respect to future research participation, the majority (75.6%) of participants indicated a preference for being interviewed in the hospital; mothers (31.3%) were more likely than fathers (16.3%) to prefer interviews at home. A large proportion of participants indicated willingness to participate in future research (99.2%), including providing saliva and blood samples (98.5% and 98.1% respectively). Those who refused cited fear of needles, personal reasons, feeling that it was unnecessary or not being interested.

Of the recruitment strategies, snowball sampling was the most effective (recruiting 46.3% of the sample), while cluster sampling was the least effective (8.3%). The other strategies each recruited about a quarter of the sample (22.1–24.1%), and 22.6% were recruited using multiple recruitment strategies. More women were recruited via direct and indirect outreaches, while more men were recruited using cluster sampling and radio adverts.

We disaggregated recruitment strategies by the number of parents present per family on account of differences in the sociodemographic characteristics between both types of families (Table 3). Snowball sampling recruited a larger proportion of single-parent households (mothers and one father). In contrast, direct outreach and cluster sampling recruited more married participants, more Muslim parents, parents with higher levels of education, and higher household wealth (for cluster sampling). There were no distinctive characteristics associated with radio adverts and indirect outreaches.

**Table 3:** Comparison of sample characteristics by recruitment strategy

Snowball		Father (n = 126)				Mother (n = 147)			
		One parent (n = 1)		Both parents (n = 125)		One parent (n = 24)		Both parents (n = 123)	
		n	%	n	%	n	%	n	%
Age	(Mean [SD])	40	–	39.5	[7.91]	34.3	[6.40]	33.0	[6.03]
Marital status	Single	1	100.0	0	0.0	10	41.7	0	0.0
	Cohabiting	0	0.0	12	9.6	3	12.5	19	15.4
	Married	0	0.0	113	90.4	11	45.8	104	84.6
Religion	Christian	1	100.0	104	83.2	22	91.7	101	82.1
	Muslim	0	0.0	21	16.8	2	8.3	11	8.9
Education	Primary	1	100.0	9	7.2	4	16.7	8	6.5
	Secondary	0	0.0	70	56.0	12	50.0	74	60.2
	Tertiary	0	0.0	43	34.4	7	29.2	40	32.5
	Postgraduate	0	0.0	3	2.4	1	4.2	1	0.8
Income (₦,000)	(Median [Range])	10	–	70	[5–5000]	20	[0–250]	20	[0–500]
Household wealth	(Mean [SD])	4.0	–	5.1	[1.59]	3.5	[1.64]	4.7	[1.69]

Direct Outreach		Father (n = 56)				Mother (n = 86)			
		One parent (n = 0)		Both parents (n = 56)		One parent (n = 2)		Both parents (n = 84)	
		n %		n	%	n	%	n	%
Age	(Mean [SD])	–	–	42.5	[7.76]	24	[0.00]	35.9	[6.16]
Marital status	Single	–	–	0	0.0	0	0.0	1	1.2
	Cohabiting	–	–	6	10.7	1	50.0	9	10.7
	Married	–	–	50	89.3	1	50.0	74	88.1
Religion	Christian	–	–	43	76.8	2	100.0	65	77.4
	Muslim	–	–	13	23.2	0	0.0	19	22.6
Education	Primary	–	–	3	5.4	0	0.0	1	1.2
	Secondary	–	–	21	37.5	1	50.0	30	35.7
	Tertiary	–	–	29	51.8	1	50.0	52	61.9
	Postgraduate	–	–	3	5.4	0	0.0	1	1.2
Income (N,000)	(Median [Range])	–	–	80	[10–500]	25	[20–30]	30	[0–900]
Household wealth	(Mean [SD])	–	–	3.9	[1.75]	3.7	[0.71]	5.2	[1.63]

Cluster sampling		Father (n = 44)				Mother (n = 5)			
		One parent (n = 0)		Both parents (n = 44)		One parent (n = 0)		Both parents (n = 5)	
		n	%	n	%	n	%	n	%
Age	(Mean [SD])	–	–	41.8	[7.59]	–	–	39.6	[5.94]
Marital status	Single	–	–	0	0.0	–	–	0	0.0
	Cohabiting	–	–	16	36.4	–	–	2	40.0
	Married	–	–	28	63.6	–	–	3	60.0
Religion	Christian	–	–	32	72.7	–	–	5	100.0
	Muslim	–	–	11	25.0	–	–	0	0.0
Education	Primary	–	–	3	6.8	–	–	0	0.0
	Secondary	–	–	24	54.5	–	–	3	60.0
	Tertiary	–	–	16	36.4	–	–	2	40.0
	Postgraduate	–	–	1	2.3	–	–	0	0.0
Income (₦,000)	(Median [Range])	–	–	200	[25–3000]	–	–	20	[20–250]
Household wealth	(Mean [SD])	–	–	5.6	[1.50]	–	–	5.0	[2.55]

Radio adverts		Father (n = 73)				Mother (n = 66)			
		One parent (n = 1)		Both parents (n = 72)		One parent (n = 3)		Both parents (n = 63)	
		n	%	n	%	n	%	n	%
Age	(Mean [SD])	40	–	41.6	[7.83]	35.0	[5.00]	33.9	[6.05]
Marital status	Single	1		0	0.0	0	0.0	1	1.6
	Cohabiting	0		10	13.9	1	33.3	10	15.9
	Married	0		62	86.1	2	66.7	52	82.5
Religion	Christian	1		58	80.6	3	100.0	51	81.0
	Muslim	0		14	19.4	0	0.0	12	19.0
Education	Primary	1		5	6.9	0	0.0	0	0.0
	Secondary	0		36	50.0	3	100.0	40	63.5
	Tertiary	0		31	43.1	0	0.0	23	36.5
	Postgraduate	0		0	0.0	0	0.0	0	0.0
Income (₦,000)	(Median [Range])	10	–	65	[10–1000[	100	[50–250]	20	[0–500]
Household wealth	(Mean [SD])	4	–	5.0	[1.75[	4.3	[1.53]	4.84	[1.43]

Indirect Outreach		Father (n = 43)				Mother (n = 87)			
		One parent (n = 0)		Both parents (n = 43)		One parent (n = 24)		Both parents (n = 63)	
		n	%	n	%	n	%	n	%
Age	(Mean [SD])	–	–	40.8	[7.18]	34.0	[5.63]	34.0	[5.76]
Marital status	Single	–	–	0	0.0	2	8.3	0	0.0
	Cohabiting	–	–	4	9.3	7	29.2	13	20.6
	Married	–	–	39	90.7	15	62.5	50	79.4
Religion	Christian	–	–	38	88.4	20	83.3	57	90.5
	Muslim	–	–	5	11.6	4	16.7	6	9.5
Education	Primary	–	–	4	9.3	4	16.7	3	4.8
	Secondary	–	–	18	41.9	9	37.5	32	50.8
	Tertiary	–	–	20	46.5	11	45.8	28	44.4
	Postgraduate	–	–	1	2.3	0	0.0	0	0.0
Income (₦,000)	(Median [Range])	–	–	50	[10–1000]	23.5	[0–120]	20	[2–500]
Household wealth	(Mean [SD])	–	–	5.12	[1.50]	4.3	[1.83]	4.3	[1.54]

### Post-Recruitment PIE

Of the 10 parents invited, seven (six mothers and one father) participated. Benefits of participation included an opportunity for their children to engage in an extra-curricular activity, which the children enjoyed. Parents who provided blood samples appreciated getting feedback of the results as an update on their physical health. This saved one mother the costs of paying for this privately, and for one father, this highlighted the need for urgent medical attention for one child. All participants acknowledged the importance of compensation for time and transport costs, which facilitated participation. Regarding dissemination, the parents suggested organizing twin festivals or celebrations which would bring households with twins together and promote future twin research. They further suggested collaborating with the traditional monarch of Ile-Ife who recently incorporated a twin festival into the naming ceremony for his own twin children. For future research, parents recommended expanding the age group of interest to increase the number of eligible households, and investigating the basis of twin differences and factors that impact on the twins’ academic performance.

## Discussion

The present study tested the feasibility of conducting twin-family research in a rural/semi-urban setting in Nigeria—a LAMI country. Three hundred and twenty families with twins were recruited using a combination of random and non-random strategies. Majority of participants were willing to participate in future twin research and provide biological samples as necessary. Snowball sampling was the most effective recruitment strategy and best for recruiting socioeconomically disadvantaged participants. In contrast, cluster sampling was the least effective and, along with direct outreach, identified participants with higher socioeconomic status. PIE activities indicated benefits experienced from participating in the study. We discuss these findings and reflect on other observations, and the implications of these for future twin-family research in South-Western Nigeria.

### Sociodemographic Characteristics of Participants

The higher proportion of those with at least secondary education in the present sample was consistent with that from a national survey (54.5–66.9%; Federal Ministry of Health and Social Welfare of Nigeria 2024). The relatively higher level of education in the present study may reflect the presence of tertiary health and education institutions at the study sites; and the tendency for research participants to be more educated than non-participants (Vo et al. 2023). The higher proportion of Christians is consistent with the distribution in Southwestern Nigeria (Jones et al. 2016); however, Muslims were underrepresented in the present study (17.9% versus 31.6–35.5%; Jones et al. 2016), possibly reflecting difficulties accessing the community. The relatively higher proportion of partnered (married or cohabiting) parents (compared to the national estimates of two-thirds, Federal Ministry of Health and Social Welfare of Nigeria, 2024) may reflect bias from initially recruiting households with both parents.

### Comparison of the Different Recruitment Strategies

Although snowball sampling was the most effective strategy, it is important to note that this strategy was augmented by the other strategies whereby members of the community who did not have twins themselves could recruit eligible contacts having heard adverts or seen fliers. This effect could also reflect the strong sense of community in relatively rural compared to more urban settings (Akindele and Adebayo 2021) and the esteem accorded twins in Yoruba culture (Leroy et al. 2002; Olupona 2023).

Two considerations which may have impacted on indirect outreaches to primary schools and religious institutions (churches and mosques) include the age of the twins in the present study. As they were mostly younger than the official age of primary school enrolment in Nigeria (6 years; Federal Republic of Nigeria 2014), indirect school outreaches would be more useful for recruiting older twins. Secondly, accessing the Muslim community was initially challenging for female field workers. This difficulty was overcome by liaising with a male Muslim cleric who himself had twin children and had participated in previous twin research. Future approaches would benefit from ensuring a sex balance among field workers to facilitate liaising with the Muslim community for recruitment.

The low yield of multi-stage cluster sampling may be linked to the eligibility criterion of having twin children. Despite the high twinning rates in Southwestern Nigeria (Akinboro et al. 2008; Smits and Monden 2011), twinning may not be common enough to lend itself to a random sampling approach. The sociodemographic profile of participants recruited using this strategy also suggests the risk for excluding marginalized groups if used exclusively for twin research.

### Sociodemographic Profile of Participants Recruited Through the Different Strategies

The high yield of fathers with direct outreach and cluster sampling suggests the importance of trusting relationships between the research team and the community for recruiting Muslim households (Stonawski et al. 2016). The higher socioeconomic status of households recruited using these strategies may reflect bias from healthcare being largely funded out-of-pocket (Madu and Osborne 2023) and membership of the cluster units (landlord associations) depending on owning houses or being longstanding tenants (e.g., Owojori and Solarin 2020).

### Post-Recruitment PIE

Parents’ willingness to participate in future research is consistent with the generally positive attitude of twins towards research (Cockburn et al. 2006; Toccaceli et al. 2009). While financial compensation facilitated participation, reported non-financial benefits including recreational outings for twins and feedback from medical investigations suggest that these can be incorporated in future studies to encourage participation (Odintsova et al. 2018). The suggested twin festivals can foster community spirit which can facilitate future twin research, although we note that these would require dedicated funding (Odintsova et al. 2018). In line with this, we have set up a system for consenting families to celebrate twins’ birthdays (telephone calls) and disseminate health-related information on a dedicated website as has been done by other twin studies (e.g., Twins Early Development Study, 2025). As advised, we are making efforts to collaborate with traditional leaders to disseminate project findings; however, the efficacy of these engagement strategies for twin research in LAMI settings need to be determined.

### Other Observations and Considerations

#### Ethical Priorities

We observed that ethical approval in the UK (but not Nigeria) emphasized the participants’ right not to know results of blood tests carried out during the study (Davies 2020). In contrast, parents requests and the child who required urgent medical attention in the present study highlighted the duty of the researcher to inform parents of results (Gordon 2009). This was illustrated in the present study when a child was discovered to be severely anemic; requiring urgent medical attention. As the family was unable to afford hospital care, we liaised with a hospital-based social worker and collaborated with the local health team including a consultant pediatrician (a member of the study team). Thus, when medical tests are being carried out as part of research in LAMI, contingency plans should be made for significant findings. These can involve working within existing local medical and social frameworks. Alternatively, a flexible/refundable budget may be incorporated in grant applications but this will depend on funders’ policies.

#### Administration of Funds

This posed a challenge as models of compensating research participants in the UK such as vouchers were not applicable in the research setting. Fund administrators in High-Income Countries (HIC) like the UK typically avoid direct participant payments to minimize fund mismanagement and prevent undue influence. However, these contrast with the priorities in LAMI countries where quick payments may be necessary to provide adequate compensation for time and the expense of travel (Bierer et al. 2021). Advanced payments by the Nigerian university (to be refunded by the UK-based institution) were not pursued because the Nigerian university (similar to many universities in LAMI countries) did not have designated research funds (Shumba and Lusambili 2021). This was eventually resolved by designating the Nigerian university a supplier after institutional checks. We recommend that HIC institutional policies be updated to recognize differing contexts of research in LAMI settings to facilitate international research collaboration.

#### Aspects of the Nigerian Sociocultural Context

Mothers sometimes required permission from their husbands to participate in the study, which reflected the patriarchal societal context which impacts on maternal and child health (Okafor et al. 2022). This also highlighted the need for getting the men’s buy-in which we did by contacting the fathers directly, having obtained their contact details from their wives. Snowball sampling also enabled men who had participated to recruit their eligible (male) contacts.

There was also a tendency for parents to under-report socially undesirable behavior in their children including behavioral symptoms such as lying/stealing or being unable to sit still. This social desirability bias may be higher in LAMI settings and lower socioeconomic classes (Ross & Mirowsky 1984; Sweetland et al. 2014) and may reflect cultural expectations of children (Nikapota 2009) whose behavior, in turn, reflect on parents and the family unit. To overcome this bias in LAMIs, research may explicitly assess social desirability (Espinosa da Silva et al. 2024), field workers trained to engage sensitively with parents, qualitative designs used to explore parents’ response motivations (Mutumba et al. 2014) and PIE activities with parents to understand how bias may be minimized.

### Strengths and Limitations

The strengths of the study include the incorporation of the whole family unit; and participant involvement which is relatively uncommon in LAMI settings (Cook et al. 2019). However, the following limitations need to be considered for the current study and twin research in LAMI settings. Individual non-random recruitment strategies may yield non-representative samples (Table 1) but our findings suggest that combining multiple strategies may overcome these individual limitations. The under-representation of single-parent households reflected our initial focus on complete families to maximize power; however, our findings indicate the need to balance this with representativeness of the sample in future research. Another factor which may have impacted on participation is the presence of other young children in the household which may make transportation difficult. While this was not specifically assessed in the present study, some parents brought the twins’ siblings with them while others came on week days when the other children could be left in school. Although we used pictures to determine the accuracy of reported zygosity in a subsample, the gold standard is genotyping (Wang et al. 2015) which can be incorporated in future research. Finally, although the present study focused on young children; our findings may not generalize to recruiting older children and adolescents. However, considering that our focus was on the family unit, we expect our findings to be relevant to twin-family research involving older children in LAMI settings.

## Conclusion

The present study demonstrated that twin-family research among pre-school children is feasible in a rural LAMI setting. Families with twins were keen to participate in research and snowball sampling (in concert with other recruitment strategies) was very efficient for recruiting households with twins. Other considerations for child and adolescent mental health research in LAMI countries include ethical priorities, funding administration, and desirability bias.

## Supplementary Information

Below is the link to the electronic supplementary material.

Supplementary file1 (DOCX 44 KB)

## Data Availability

The data that support the findings of this study are available from Wolfson Centre for Young People's Mental Health, but restrictions apply to the availability of these data, which were used under licence for the current study and so are not publicly available. The data are, however, available from the authors upon reasonable request and with the permission of Wolfson Centre for Young People's Mental Health.
