# Supplementary material for: Child Mental Health Research in Low- and Middle-Income Countries: A Twin-Family Feasibility Study in Nigeria
Source: Behav Genet. 2025 Oct 13;55(6):438–53. doi: 10.1007/s10519-025-10235-z (PMC12719352; doi:10.1007/s10519-025-10235-z)
Supplement: Supplementary file 1 — Supplementary file1 (DOCX 44 KB) [file 10519_2025_10235_MOESM1_ESM.docx]

Supplementary Material for

**Child mental health research in Low- and Middle-Income Countries: A twin feasibility study in Nigeria**

Olakunle Ayokunmi Oginni^1,^(0000-0002-2340-9429), Olatokunbo Oguns^2^, Olusola Jeje^3^ (0000-0002-2765-8158), Oluwatosin Olorumoteni^4^ (0000-0001-8561-9918), Boladale Mapayi^5^ (0000-0002-0596-2132), Ruth Gilbert^6^ (0000-0001-9347-2709), Dan J. Stein^7^ (0000-0001-7218-7810), Frühling Rijsdijk^8^ (0000-0003-4762-2803), Anita Thapar^1^ (0000-0002-3689-737X)

1. Wolfson Centre for Young People’s Mental Health and Centre for Neuropsychiatric Genetics and Genomics, Division of Psychological Medicine and Clinical Neuroscience, Cardiff University, United Kingdom

2. Department of Haematology, Obafemi Awolowo University, Ile-Ife, Nigeria

3. Department of Chemical Pathology, Obafemi Awolowo University, Ile-Ife, Nigeria

4. Department of Paediatrics, Obafemi Awolowo University, Ile-Ife, Nigeria

5. Department of Mental Health, Obafemi Awolowo University, Ile-Ife, Nigeria

6. University College London Institute of Child Health, University College London, United Kingdom

7. SAMRC Unit on Risk & Resilience in Mental Disorders, Department of Psychiatry and Neuroscience Institute, University of Cape Town, South Africa

8. Department of Psychology, Anton de Kom University, Suriname

| **Supplementary Table 1:** Summary of constructs assessed and questionnaires used | | | | |
| --- | --- | --- | --- | --- |
|  |  | |  | |
|  | **Parents** | | **Children** | |
| **Construct (Variable category)** | **Variables** | **Questionnaire** | **Variables** | **Questionnaire** |
| **Verification^a^** | i. Identity check | - National Identity Number and photo identification | i. Identity check | - Birth certificates - Photographs of twins and parents |
| **Sociodemographic** | i. Age, sex, marital  status, religion | - Single question each | i. Sex, date of birth | - Single question each |
|  | ii. Socioeconomic status | - Single questions to assess highest level of education, occupation, household wealth^[1]^ | ii. Zygosity | - Questions to assess physical similarity between twins^[2]^ |
| **Feasibility** | i. How participants  heard about the study | - Single question with 5 recruitment strategies as   options | - | - |
|  | ii. Preferred site of data collection | - Single questions with options of home versus hospital |  |  |
|  | iii. Date of data  collection | - Single question |  |  |
|  | iv. Willingness to participate in future longitudinal twin  research | - Single questions with Yes/No options |  |  |
|  | v. Willingness to provide blood and saliva samples for self and children in present and future studies | - Separate single questions with Yes/No options | - | - |
|  | vi. Preferred means of future of future  contact | - Single question with multiple response options |  |  |
|  | vii. Reasons for *iv*. and *v*. above | - Open ended questions linked to above |  |  |
| **Psychopathology/ Psychological** | i. Depressive symptoms | - PHQ-9^[3]^ | i. Emotional symptoms | - SDQ[4] |
|  | ii. Anxiety symptoms | - GAD-7^[5]^ | ii. Hyperactivity- impulsivity symptoms | - SDQ[4] |
|  | iii. Alcohol use | - AUDIT-C^[6]^ | iii. Behavioral (Conduct) symptoms | - SDQ[4] |
|  |  |  | iv. Autism spectrum symptoms | - Checklist for autism in toddlers^[8]^ |
|  | iv. ADHD symptoms | - WHO ADHD Self-Report Screening Scale^[9]^ |  |  |
|  | v. Lifetime psychiatric diagnoses | - Single question with multiple response options |  |  |
|  |  |  |  |  |
| Supplementary Table 1 (continued) | | | | |
|  | **Parents** |  | **Children** |  |
| **Construct (Variable category)** | **Variables** |  | **Variables** | **Questionnaire** |
| **Biological factors: Physical health** | i. Chronic medical conditions | - CIDI checklist^[10]^ | i. Medical conditions | - Checklist of chronic illnesses (e.g., sickle disease, epilepsy and asthma) and frequency of acute illnesses in past 6 months |
|  | ii. Malnutrition and General physical health | - Anthropometric measurements (Height and weight), serum albumin^b^; Full blood count^c^ | ii. Malnutrition and General physical health | - Anthropometric measurements (Height, weight, mid-upper arm circumference), serum albumin^b^; Full blood count^c^ |
|  | iii. Inflammatory markers | - C-Reactive Protein^d^, - Tumour Necrosis Factor-α^e^, Interleukin-6^f^ | iii. Inflammatory markers | - C-Reactive Protein^d^, Tumour Necrosis Factor-α^e^, Interleukin-6^f^ |
|  |  |  | iv. Pregnancy and delivery history | - Single questions |
| **Social factors: Home environment/Child’s characteristics** | i. Domestic violence | - HARK questionnaire^[11]^ | i. Physical and emotional abuse/neglect | - Questions selected from the WHO ACE- International Questionnaire^[12]^ |
|  | ii. Chaotic home environment | - Confusion, Hubbub and Order Scale^[13]^ | ii. Bully victimisation | - Questions adapted from the E-Risk study^[31]^ |
|  | iii. Incarceration | - Single question | iii. Childhood gender nonconformity | - Parent-Report Gender Identity questionnaire for Children^[14]^ |
|  | iv. Stressful life events | - Questions selected from a list of stressful life events | iv. Irritability | - Affective Reactivity Index^[16]^ |
|  | v. Mental health stigma | - Bogardus social distance scale^[17]^ | v. Harsh parenting | - Parental Discipline Questionnaire^[18]^ |
|  | vi. Childhood gender nonconformity | - Recalled Childhood Gender identity Questionnaire^[19,^ ^20]^ |  |  |
| **Protective factors** | i. Resilience | - Connor-Davidson Resilience scale^[21]^ | i. Parental warmth | - Parental Feelings questionnaire^[22]^ |
|  | ii. Social connectedness | - Social connectedness scale- Revised^[23]^ | ii. Cognitive ability | - Molteno Developmental Scale^[24]^ |
|  | iii. Religiosity | - Religiosity orientation test ^[25]^ |  |  |

*Note.* ^a^These were to confirm eligibility; ^b^serum albumin were assessed using the Cobas c311 analyser^[26]^; ^c^Full blood count were carried out using the Mindray BC-10 three-part Auto Haematology Analyser^[27]^; ^d^C-Reactive Protein, ^e^Tumour Necrosis Factor-α and ^f^Interleukin-6 were assessed using the hs-CRP (highly sensitive CRP) AccuBind^[28]^, the Human Tumor Necrosis Factor Αlpha (TNF-A)^[29]^ and the Human Interleukin 6 (IL-6)^[30]^ Enzyme-Linked Immunosorbent Assay (ELISA) kits respectively.

ACE=Adverse Childhood Experiences; ADHD=Attention Deficit Hyperactivity Disorder; AUDIT=Alcohol Use Disorder Identification Test; CIDI= Composite International Diagnostic Interview for DSM-IV; GAD- 7=7-item Generalised Anxiety Questionnaire; PHQ-9=Patient Health Questionnaire; SDQ=Strengths and Difficulties Questionnaire; WHO=World Health Organisation.

**References**

1. Jayaweera K, Aschan L, Pannala G, Adikari A, Glozier N, Ismail K, et al. The Colombo Twin and Singleton Follow-up Study: A population based twin study of psychiatric disorders and metabolic syndrome in Sri Lanka. BMC Public Health 2018; 18: 1-15. https://doi.org/10.1186/s12889-017-4992-2

2. Goldsmith HH. A zygosity questionnaire for young twins: A research note. Behavior Genetics 1991; 21: 257-269. https://doi.org/10.1007/BF01065819

3. Kroenke K, Spitzer RL, Williams JB. The PHQ-9: validity of a brief depression severity measure. Journal of General Internal Medicine 2001; 16: 606-613. https://doi.org/10.1046/j.1525-1497.2001.016009606.x

4. Goodman R. The Strengths and Difficulties Questionnaire: A research note. Journal of Child Psychology and Psychiatry 1997; 38: 581-586. https://doi.org/10.1111/j.1469-7610.1997.tb01545.x

5. Spitzer RL, Kroenke K, Williams JB, Löwe B. A brief measure for assessing generalized anxiety disorder: The GAD-7. Archives of Internal Medicine 2006; 166: 1092-1097. https://doi.org/10.1001/archinte.166.10.1092

6. Bush K, Kivlahan DR, McDonell MB, Fihn SD, Bradley KA. The AUDIT alcohol consumption questions (AUDIT-C): an effective brief screening test for problem drinking. Archives of Internal Medicine 1998; 158: 1789-1795. https://doi.org/10.1001/archinte.158.16.1789

7. Berman AH, Bergman H, Palmstierna T, Schlyter F. DUDIT − The Drug Use Disorders Identification Test, MANUAL Version 1.0. Stockholm: Karolinska Institutet, Department of Clinical Neuroscience, Section for Alcohol and Drug Dependence Research; 2003.

8. Haworth CM, Davis OS, Plomin R. Twins Early Development Study (TEDS): a genetically sensitive investigation of cognitive and behavioral development from childhood to young adulthood. Twin Research and Human Genetics 2013; 16: 117-125. https://doi.org/10.1017/thg.2012.91

9. Ustun B, Adler LA, Rudin C, Faraone SV, Spencer TJ, Berglund P, et al. The World Health Organization adult attention- deficit/hyperactivity disorder self-report screening scale for DSM-5. JAMA Psychiatry 2017; 74: 520-526. https://doi.org/10.1001/jamapsychiatry.2017.0298

10. Kessler RC, Abelson J, Demler O, Escobar JI, Gibbon M, Guyer ME, et al. Clinical calibration of DSM-IV diagnoses in the World Mental Health (WMH) version of the World Health Organization (WHO) Composite International Diagnostic Interview (WMH-CIDI). International Journal of Methods in Psychiatric Research 2004; 13: 122-139. https://doi.org/10.1002/mpr.169

1. Sohal H, Eldridge S, Feder G. The sensitivity and specificity of four questions (HARK) to identify intimate partner violence: a diagnostic accuracy study in general practice. BMC Family Practice 2007; 8: 49. https://doi.org/10.1186/1471-2296-8-49

12. Pace CS, Muzi S, Rogier G, Rogier G, Meinero LL, Marcenaro S. The Adverse Childhood Experiences–International Questionnaire (ACE-IQ) in community samples around the world: A systematic review (part I). Child Abuse and Neglect 2022; 129: 105640. https://doi.org/10.1016/j.chiabu.2022.105640

13. Wong MD, Sarkisian CA, Davis C, Kinsler J, Cunningham WE. The association between life chaos, health care use, and health status among HIV-infected persons. Journal of General Internal Medicine 2007; 22: 1286-1291. https://doi.org/10.1007/s11606-007-0265-6

14. Johnson LL, Bradley SJ, Birkenfeld-Adams AS, Radzins-Kuksis MA, Maing DM, Mitchell JN, et al. A parent-report gender identity questionnaire for children. Archives of Sexual Behavior 2004; 33: 105-116. https://doi.org/10.1023/B:ASEB.0000014325.68094.f3

15. Olusakin A. The Relationship between Stressful Life Events and Gender among Nigerians. Lagos Journal of Educational Research 2004; 2: 56-66. https://ir.unilag.edu.ng/handle/123456789/6071

16. Stringaris A, Goodman R, Ferdinando S, Razdan V, Muhrer E, Leibenluft E, et al. The Affective Reactivity Index: A concise irritability scale for clinical and research settings. Journal of Child Psychology and Psychiatry 2012; 53: 1109-1117. https://doi.org/10.1111/j.1469-7610.2012.02561.x

17. Bogardus ES. Measuring social distance. Journal of Applied Sociology 1925; 9: 299-308.

18. Deater-Deckard K. Parenting and child behavioral adjustment in early childhood: A quantitative genetic approach to studying family processes. Child Development 2000; 71: 468-484. https://doi.org/10.1111/1467-8624.00158

19. Alanko K, Santtila P, Harlaar N, Witting K, Varjoen M, Jern P, et al. Common genetic effects of gender atypical behavior in childhood and sexual orientation in adulthood: A study of Finnish twins. Archives of Sexual Behavior 2010; 39: 81-92. https://doi.org/10.1007/s10508-008-9457-3

20. Zucker K, Milhausen R, Sakaluk J, Fisher T. Recalled Childhood Gender Identity/Gender Role Questionnaire. In Milhausen RR, Sakaluk JK, Fisher TD, Davis CM, Yarber WL. (eds) Handbook of Sexuality-Related Measures. New York and London: Routledge, Taylor and Francis Group; 2019.

21. Aloba O, Olabisi O, Aloba T. The 10-item Connor–Davidson Resilience Scale: Factorial structure, reliability, validity, and correlates among student nurses in southwestern Nigeria. Journal of the American Psychiatric Nurses Association 2016; 22: 43-51. https://doi.org/10.1177/1078390316629971

22. Deater–Deckard K, Dodge KA, Bates JE, Pettit GS. Multiple risk factors in the development of externalizing behavior problems: Group and individual differences. Development and Psychopathology 1998; 10: 469-493. https://doi.org/10.1017/S0954579498001709

23. Lee RM, Robbins SB. Measuring belongingness: The social connectedness and the social assurance scales. Journal of Counseling Psychology 1995; 42: 232-241. https://doi.org/10.1037/0022-0167.42.2.232

24. Honeth I, Laughton B, Springer PE, Cotton MF, Pretorius C. Diagnostic accuracy of the Molteno Adapted Scale for developmental delay in South African toddlers. Paediatrics and International Child Health 2019; 39: 132-138. https://doi.org/10.1080/20469047.2018.1528754

25. Idehen EE. The development and evaluation of a religiosity scale. IFE Psychologia: An International Journal 2001; 9: 58-69. https://hdl.handle.net/10520/EJC38934

26. Hoffmann-La Roche Ltd. cobas c 311 analyzer. Available from: https://diagnostics.roche.com/global/en/products/instruments/cobas-c-311-ins-2043.html [Accessed 20th August 2023].

27. Mindray. Auto Hematology Analyzer BC-10. Available from: https://[www.mindray.com/en/products/laboratory-](http://www.mindray.com/en/products/laboratory-) diagnostics/hematology/3-part-differential-analyzers/bc-10 [Accessed 20th August 2023].

28. Monobind Inc. hs-CRP Monobind Inc. Available from: https://[www.monobind.com/Products/immunoassays-cardiac-](http://www.monobind.com/Products/immunoassays-cardiac-) markers-hs-crp [Accessed 20th August 2023].

29. Bioassay Technology Laboratory. Human Tumor Necrosis Factor Αlpha, TNF-A ELISA Kit Available from: https://www.bt- laboratory.com/index.php/Shop/Index/productShijiheDetail/p_id/247/cate/kit.html [Accessed 20th August 2023].

30. Bioassay Technology Laboratory. Human Interleukin 6, IL-6 ELISA Kit: Bioassay Technology Laboratory. Available from: https://[www.bt-laboratory.com/index.php/Shop/Index/productShijiheDetail/p_id/253.html](http://www.bt-laboratory.com/index.php/Shop/Index/productShijiheDetail/p_id/253.html) [Accessed 20th August 2023].

31. Bowes L, Maughan B, Caspi A, Moffitt TE, Arseneault L. Families promote emotional and behavioural resilience to bullying: Evidence of an environmental effect. Journal of Child Psychology and Psychiatry 2010; 51: 809-817. https://doi.org/10.1111/j.1469-7610.2010.02216.x
